# Polar code construction by estimating noise using bald hawk optimized recurrent neural network model

**DOI:** 10.1038/s41598-025-07886-7

**Published:** 2025-07-02

**Authors:** Sunil Yadav Kshirsagar, Venkatrajam Marka

**Affiliations:** Department of Mathematics, School of Advanced Sciences, VIT-AP University, Beside AP Secretariate, Amaravati, Andhra Pradesh 522241 India

**Keywords:** Recurrent neural network (RNN), Polar code construction, Noise estimation, Bit error rate (BER), Frame error rate (FER), Engineering, Mathematics and computing

## Abstract

Polar codes are making significant progress in error-correcting coding due to their ability to reach the limit of the Shannon capacity of communication channels, indicating great advancements in the field. Decoding errors are common in real communication channels with noise. The main objective of this study is to develop a recurrent neural network decoder for robust polar code construction with the Bald Hawk Optimization (RNN-based Decoder with BHO) model that can estimate the error in information bits. This research presents a practical and significant innovation by combining recurrent neural networks (RNNs) for noise estimation in polar coding with a Bald Hawk optimization approach. Moreover, this synthesis of RNN-based noise estimation with Bald Hawk optimization makes the polar coding system more flexible and adaptive, allowing for more accurate noise estimation during decoding. In terms of frame errors, the Bit Error Rate (BER), Binary Phase Shifting Key-BER (BPSK-BER), and Frame Error Rate (FER) achieve the lowest error values of 0.0000087, 0.01519, and 0.000182, respectively. Similarly, in a 4 dB SNR context, the BER, BPSK-BER, and FER achieve values of 0.0000073, 0.02065, and 0.000108, respectively. The results shows that the proposed RNN-based decoder with BHO model outperforms the existing decoders.

## Introduction

Before generating the polar code, one must first calculate the communication channel’s capacity. The maximum data rate that a channel can safely transmit depends on its capacity. Understanding channel capacity is crucial to creating effective polar codes. The author formulates the design criteria after estimating the channel capacity to achieve the desired performance^1–5^. Design criteria include the desired coding rate, error correction if necessary, and special considerations for specific communication requirements. The basic concept behind polar codes is based on channel polarization. Channel polarization is a phenomenon that divides several sub-channels into more reliable (frozen) and less reliable (information) channels^6–9^. On the other hand, the bit-channel mapping process assigns fixed values to more reliable (frozen) channels, which correspond to the desired coding rate, and assigns bits to less reliable information channels. To form a polar codeword, the information bits should merge with the frozen bits. Successive cancellation (SC) decoding is one of the polar code’s key features. It is a decoding mechanism that accurately determines the transmitted information in the form of a noisy received signal^10^. You can tune polar codes to specific coding rates and block sizes^11–14^.

For instance, one can either shorten or puncture a polar code to accommodate variable-length messages with non-integer coding rates. The author evaluates the generated polar code’s performance using simulated operations on a specific communication channel model^15^. To evaluate the performance of a code, variables such as bit error rate (BER) and frame error rate (FER) are used. Iterative refinement can be used during the polar code generation process to increase code efficiency^16^. To achieve high-speed and efficient decoding for practical applications, polar codes are frequently implemented in hardware using application-specific integrated circuits (ASICs) or field-programmable gate arrays (FPGAs)^17,18^. Polar codes easily adapt to different coding rates, allowing customization for different communication requirements. They are advantageous in many situations because they can efficiently support high and low code rates^19–21^. Polar codes can be effectively used during the decoding process to benefit hardware implementations from parallel computing. Parallel decoding allows for faster and more efficient data retrieval^22,23^. Recently, various decoders for polar codes have been developed using deep-learning approaches. They are advantageous in faster communication in polar codes^24–26^. Belief Propagation (BP) decoders had low latency and utilized an early stopping mechanism to reduce the decoding complexity. Early Stopping Belief Propagation (ESBP) based model reduces the average number of iterations within the decoder to improve the decoding efficiency and performance of the model. The decoding ability of the ESBP decoder for polar codes has higher efficiency than the conventional ESBP decoders^31^.

This research presents an innovative recurrent neural network-based decoder for robust polar code construction with Bald Hawk Optimization (RNN-based Decoder with BHO) model that can estimate the error in information bits. Bald hawk optimization uses cooperative hunting strategies of Harris hawks with focused hunting techniques by bald eagles for tuning with RNN classifiers. This research deals with the effect of noise on data transmission as a means of increasing efficiency and reliability for communication systems. The study attempts to provide a more reliable and accurate communication model by combining noise estimation through RNN and polar coding. This is very important when noise degrades the accuracy of the transmitted data, which requires adjustments to the encoding and decoding techniques so as not to degrade this interference. Therefore, this hybridization is crucial for effective and adaptive noise estimation in polar coding systems. Using RNNs, sequential dependencies in noisy communication channels are captured, and Bald Hawk optimization fine-tunes the actions of these networks to adequately handle dynamic noise patterns. Furthermore, these two aspects will guarantee a more effective polar coding method that provides higher noise estimation accuracy and more reliability in the decoding process. The major contributions involved in this research are as follows,

- **Bald Hwak Optimization (BHO):** This hybrid optimization is based on the merge of the Harris Hawk chasing method and bald eagle hunting behavior. A hybrid algorithm will take the form of collaborative exploitation where solutions work together to navigate the solution space in an agile and efficient manner as illustrated in Hawkes’ trick.
- **RNN-based decoder with BHO Model:** The RNN-based decoder with the BHO model to learn noise patterns enables the prediction of noise properties in the received signal. An optimization using RNN-based polar coding calculates the error rate of noisy channels. The proposed decoder makes communication more robust and effective by changing encoding or decoding noise levels beyond that threshold. This hybrid approach combined with RNNs’ adaptability helps effective error correction in the process of polar decoding.

This manuscript outlines the methodology of the study, which is structured as follows: Section 2 discusses previous techniques of polar code generation along with their pros and cons. The proposed polar code generation model in Section 3 and Section 4 discusses in detail the Bald Hawk optimization model. Finally, Section 5 shows the effectiveness of the obtained results and Section 6 presents a comprehensive outline of all obtained results.

## Literature review

Marvin Geisel Hart et al.^3^ proposed an enhanced version of the iterative belief propagation list (BPL) decoding algorithm, unlike simple error-detection techniques. This algorithm incorporates CRC for error correction instead of simple error detection, resulting in improved accuracy. However, estimating prior channel characteristics can be challenging because practical communication situations suffer from noise, and incorrect channel estimation can lead to poor performance and poor channel estimation.

In their study, Zheng et al.^4^ presented a new approach to polar coding for unsourced, uncoordinated Gaussian random access channels. This method has shown excellent performance in high-user density regions and also demonstrated satisfactory competitiveness in low- and medium-user density regions. Polar codes support multiple code rates but it is very challenging to modify the code rate based on the communication conditions in real-time.

Instead of using an SC decoding approach that leads to larger decode latency, Ahmed Elkelesh et al.^5^ designed a new polar code construction for arbitrary channels and optimized it specifically for an individual decoder algorithm. However, since the rate of spread of polarization is limited among channels, they may decelerate.

Yong Fang et al.^6^ developed three adaptive algorithms, in which an estimated channel position is initially given and improved with each iteration. The result was exceptional accuracy compared to previous models. But, especially when there are channel fading or errors in transmission, it is challenging to find the alignment word of the polar code at the receiving end.

Xinjin Lu et al.^7^ developed a hybrid physical-layer encryption and peak-to-average power ratio (PAPR) mitigation strategy to solve the PAPR problem, thereby providing high security in transmission systems. Moreover, this method has helped to reduce PAPR in OFDM systems. However, it is difficult to construct robust polar codes in real-time systems with high latency requirements considering hardware limitations and timing constraints.

Moustafa Ebada et al.^8^ elaborated the method of decoding polar codes using BP decoders in a multiuser setting. This has helped to achieve a good error rate of decoding complexity while achieving the highest possible rate of flexibility. But at the same time with the addition of feedback mechanisms, generating polar codes can become a more challenging and complex process that requires efficient algorithms to set up effective feedback.

Bi He et al.^27^ designed a machine learning-based multi-flip SC decoding algorithm to simultaneously enhance the performance of SCF decoding and provide optimal implementation. However, it is difficult to predict which set of frozen bits will achieve performance goals without low decoding complexity.

Md abdul aziz et al.^32^ designed a Bidirectional long short-term memory (Bi-LSTM) based decoder model, which processes sequences in forward and backward directions to progress the polar-coded short packet transmission over a flat fading channel. Although this approach performed well with high modulations compared with CNN and DNN, lost reliability at higher SNR values for effective decoding. The Bi-LSTM-based model failed to decode the polar codes effectively.

### Challenges

- Training RNNs to efficiently learn channel properties and reliably decode code words often requires relatively large amounts of data. Training with large datasets can be a time-consuming task, especially when there is a lack of specific communication environments or useful data^2^.
- RNNs decode bits one after another sequentially, and errors introduced in early bit judgments can propagate through the entire decoding process till they influence decision judgment for other remaining bits. This error propagation problem may affect the overall decoding performance^3^.
- Most models are prone to over-fitting, especially when the training data is sparse. Over-fitting occurs when a model learns a particular data set without generalizing it to new data, which reduces the performance of the actual communication channel^4^.
- Standard RNNs may fail to decode the entire codeword due to their memory limitations. As a result, decoding accuracy and overall performance may deteriorate, especially under noise and interference conditions^6^.
- The Bi-LSTM-based decoder model struggled to decode the polar codes effectively. The higher-order modulations in the Bi-LSTM-based model affected the generalization and increased complexity^32^.

The above-mentioned limitations are overcome by the utilization of advanced methods in the proposed model. Bald hawk optimization is employed to enhance effective polar code encoding with low latency and robustness in communication. The incorporation of Harris Hawk chasing methods and Bald Eagle hunting behavior provides a chase strategy and focusing vision with hunting technique. The hybrid optimization combines the polar coding technique with RNNs improving the adaptability for effective noise management and better generalization. This approach combines the advantages of RNNs in noise estimation with the efficiency and reliability provided by polar encoding in communication which is explained in this research below.

## Methodology

**Fig. 1:**
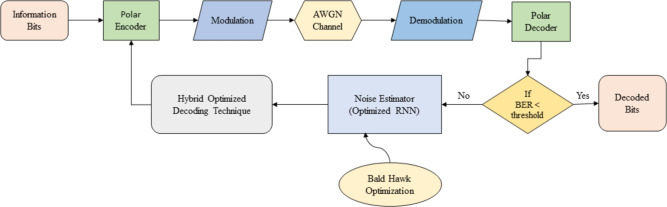
Architecture of the proposed polar code construction model.

The primary objective of the research is to develop a high-performance polar code constructor by accurately estimating the noise in the information bits. At the beginning, the polar encoder takes the information bits as input. Its purpose is to transform a set of input bits into a longer set of encoded bits using a specific encoding method called polar encoding. The polar encoder is essential for achieving efficient and reliable communication in modern systems. Polar encoding is a robust error-correcting coding technique that effectively reaches the capacity limit of binary input symmetric memoryless channels. Once the polar encoding is completed, the data will undergo modulation to convert the encoded bits into a format suitable for transmission through the communication channel. Modulation is the process that maps the digitally encoded bits to an analog waveform that can be transmitted over the channel. The AWGN (Additive White Gaussian Noise) channel will receive the modulated output, replicating the noise effects in communication systems. The noisy bits are then transferred to demodulation to convert analog waveform into digital form. Subsequently, the polar decoder will retrieve the original transmitted information from the encoded bits received in a polar coding system. The decoding process plays a vital role in ensuring dependable and precise communication. If the achieved Bit error rate falls below the threshold, then the decoded bits will be obtained. However, if the bit error rate exceeds the threshold, noise estimation will be carried out using an optimization-enabled recurrent neural network. The utilization of an RNN classifier in polar encoding for noise estimation refers to employing an RNN model to forecast the noise characteristics, specifically in the context of polar coding. This approach combines the advantages of RNNs in noise estimation with the efficiency and reliability provided by polar encoding in communication. Figure 1 illustrates the proposed framework, which utilizes the hybridized features of the bald eagle and Harris hawk to enable the Bald hawk optimization. This optimization technique effectively tunes the classifier. By incorporating a noise estimator, the framework accurately estimates the noise characteristics in the received signal. This information is then used to improve the performance of the subsequent decoding process. Finally, the hybridized decoding technique is used to obtain the updated information bits and then again the process repeats. This process keeps repeating until we get the desired decoded output.

### Preliminary phase

In the early stages of research, it is necessary to assess the level of loudness regarding bits of information. To proceed with the encoding and modulation process, it is critical to determine what level of noise can complicate or interfere with the data transmission and decoding. We refer to these random disturbances or oscillations as noise when they impact the transmitted data. Noise estimation also involves monitoring channel or received signal characteristics to identify potential interference or other disturbances that may affect communication quality. This estimate allows for a better understanding of the predicted level of interference and the implementation of efficient encoding and decoding strategies that minimize the effects of noise.

### Encoding of polar codes

A binary polar code can be described as a specific type of linear block code by using the notation $(T, M, \delta , c^{\delta })$. Here, *T* represents the block length which is equal to $2^{p}$. *M* is the number of information bits encoded for each code word. The set of indices for the frozen bits positions, denoted by $\delta$, is chosen from $\{1,2,...,T\}$ and $c^{\delta }$ is a vector containing the frozen bits. Both the encoder and the decoder know the fixed binary sequence used to assign the frozen bits.

A generator matrix is used to perform the encoding operation for a vector of information bits, *C*, in a $(T, M, \delta )$ polar code. The generator matrix $K_{T}$ is expressed as $K_{T} = K_{2}^{\otimes p}$, where $$K_{2}= \begin{bmatrix} 1 & 0\\ 1 & 1 \\ \end{bmatrix}$$ and $\otimes$ is the Kronecker product. The codewords are generated using the data sequence *Q*, shown in equation 1 as follows,

1 $$\begin{aligned} Q = HK_{T} = {H^{\delta }}^{t}(K_{p})_{\delta ^{t}} + H^{\delta }(K_{p})_{\delta } \end{aligned}$$

where the indices $\delta ^{t} = \{1,2,...,T\}/\delta$ represent the indices of the non-frozen bits. The data sequence is denoted as ${H^{\delta }}^{t}$, while the frozen bits, typically assigned a value of zero, are represented by $H^{\delta }$.

Polar encoding is a basic process in coding theory that tries to convert a block of input information bits into a longer sequence of encoded bits. The purpose of polar encoding is to improve data transmission reliability using error correction and detection mechanisms. This technique exploits the effect of polarization, where certain channels become noisy or noiseless based on their characteristics. The polar encoding process applies a systematic transformation to the original information bits. The transformation involves operations with bits, permutations, and copies of the sequence to produce a new series of coded elements. To ensure proper encoding, the indices of the input vector should be bit-reversed. The encoding technique selectively combines the input bits to make use of features that a polarized channel offers. This therefore means that some bits are more reliable in transmission and detection, while others can have errors. The encoded bits carry redundancy information, enabling the receiver to rectify errors or detect corrupted bits. We deliberately arrange this redundancy to enhance the likelihood of correct bit recovery. Polar encoding establishes the foundation upon which reliable and efficient communication is possible, especially when using noisy channels.

### Modulation

Following polar encoding, encoded bits are used as input for subsequent processing, such as modulation and channel transmission. By coupling this encoding method with subsequent stages in the communication chain, it is possible to achieve reliable and error-resistant data transport via modern communication systems. During transmission, modulation transforms digital bits into the waveform that encodes analog signals. We can multiplex modulated signals because they are less susceptible to noise. In our study, modulation changes encoded bits for successful transmission across the communication channel.

### AWGN channel simulation

A type of random noise known as AWGN frequently disrupts communication networks. In the AWGN channel simulation, we add AWGN noise to the transmitted waveform signal to replicate real-world conditions. This simulation replicates the noise’s impact on the signal and helps determine system performance at real-world noise levels. It’s important to determine whether the approach handles transmission noise well.

### Demodulation

Following AWGN channel simulation, the demodulation process uses noisy bits as input for subsequent processing. During transmission, demodulation converts waveform signals into digital signals to provide better input for the decoder.

### Decoding of polar codes

During polar decoding, complex algorithms examine the received demodulated bits to reverse the encoding process. As a result, we determine the best order to work with the received data and learn the decoding method effectively. Initially, this process involves deciding on the bit values to transmit. Polar decoding uses error correction techniques to correct transmission-related errors. The goals include accurate information retrieval and restoring the integrity of transmitted data. This is crucial when noise or interference corrupts the transmitted data, enabling effective communication. Our investigation reveals that polar decoding correctly converts the encoded bits to their original information bits.

Polar codes, when decoded with the successive cancellation (SC) decoding algorithm, can achieve channel capacity in code length as the length of the code approaches infinity. The SC decoding algorithm estimates the bits $\hat{c}_{q}$ in a sequential manner, where *q* ranges from $0\;\le \;q\;\le \;T$. The estimation of $\hat{c}_{q}$ is dependent on the modulated output $d^{T}$ and the previous bit decisions $\hat{c}_{1},\hat{c}_{2},...,\hat{c}_{i-1}$, represented as $\hat{c}_{1}^{q-1}$. The polar decoder applies specific rules in estimating $\hat{c}_{q}$ as shown in equation 2

2 $$\begin{aligned} \hat{c}_{q}={\left\{ \begin{array}{ll} c_{q}, & if \, q \, \in \, \delta \\ ~\\ 0, & if \, q \, \in \, \delta ^{t} \, \& \, D\left( d_{1}^{T}, \hat{c}_{1}^{q-1}\right) \, \ge \, 1 \\ ~\\ 1, & if \, q \, \in \, \delta ^{t} \, \& \, D\left( d_{1}^{T}, \hat{c}_{1}^{q-1}\right) \, < \, 1. \end{array}\right. } \end{aligned}$$

The probability of a non-frozen bit can be determined by calculating the $q^{th}$ likelihood ratio (LR) $D_{T}^{q}\left( d_{1}^{T}, \hat{c}_{1}^{q-1}\right)$ at length *T*, which can be computed recursively using two formulas as shown in equation 3 and equation 4.

3 $$\begin{aligned} & D_{T}^{2q-1}\left( d_{1}^{T}, \hat{c}_{1}^{2q-2}\right) = \frac{D_{T/2}^{q}\left( d_{1}^{T/2}, \hat{c}_{o}^{2q-2}\oplus \hat{c}_{y}^{2q-2} \right) D_{T/2}^{q}\left( d_{1}^{{T/2}+1}, \hat{c}_{y}^{2q-2} \right) + 1}{D_{T/2}^{q}\left( d_{1}^{T/2}, \hat{c}_{0}^{2q-2}\oplus \hat{c}_{y}^{2q-2} \right) + D_{T/2}^{q}\left( d_{1}^{{T/2}+1}, \hat{c}_{y}^{2q-2} \right) } \end{aligned}$$

4 $$\begin{aligned} & D_{T}^{2q-1}\left( d_{1}^{T}, \hat{c}_{1}^{2q-2}\right) = D_{T/2}^{q}\left( d_{1}^{T/2}, \hat{c}_{o}^{2q-2}\oplus \hat{c}_{y}^{2q-2} \right) ^{1-2\hat{c}_{2q-1}} D_{T/2}^{q}\left( d_{1}^{{T/2}+1}, \hat{c}_{y}^{2q-2} \right) \end{aligned}$$

The symbols $\hat{c}_{o}^{2q-2}$ and $\hat{c}_{y}^{2q-2}$ represent the parts of $\hat{c}_{0}^{2q-2}$ that have odd and even indices respectively. Therefore, to calculate the LR at length *T*, we can calculate two LRs at length *T*/2 and then break them down recursively to a block length of *T*. The initial LRs can be determined from the channel observation. Due to the high cost of implementing multiplication and division operations in hardware, these operations are often avoided and instead performed in the logarithm domain using the functions $\beta$ and $\eta$ mentioned as in equation 5, 6 and equation 7 as follows:

5 $$\begin{aligned} & \beta (D_{1},D_{2}) = 2 \tanh ^{-1}\left[ \tanh \left( \frac{D_{1}}{2}\right) \tanh \left( \frac{D_{2}}{2}\right) \right] \end{aligned}$$

6 $$\begin{aligned} & \beta (D_{1},D_{2}) \approx sign(D_{1}D_{2})min(|D_{1}||D_{2}|) \end{aligned}$$

7 $$\begin{aligned} & \eta (D_{1},D_{2}) = (-1)^{1-2\hat{c}_{2q-1}} D_{1} + D_{2} \end{aligned}$$

where $D_{1} = \log \left[ D_{T/2}^{q}\left( d_{1}^{T/2}, \hat{c}_{o}^{2q-2}\oplus \hat{c}_{y}^{2q-2} \right) \right]$ and $D_{2}=\log \left[ D_{T/2}^{q}\left( d_{1}^{{T/2}+1}, \hat{c}_{y}^{2q-2}\right) \right]$ are log-likelihood ratios (LLRs). In practical implementations, the minimum function can be used to approximate the function $\beta$, according to equation 7.

### Threshold-based bit error rate analysis

BER analysis is an approach that checks the accuracy of bits transmitted and received in order to assess how a communication system works. The bit error rate shows the ratio of erroneous bits to all transmitted bits. In threshold-based BER analysis, the system’s validity hinges on whether the BER falls below a predetermined threshold. Therefore, we often choose the threshold based on our tolerance for system error. If the actual bit error rate is less than a predetermined threshold, the transmission shall be considered a success. If the system surpasses the threshold, it might not provide performance-matching output. The BER analysis based on a threshold provides important information about the system’s ability to handle noise, interference, and other factors that may affect the accuracy of transmitted bits. We have used threshold-based BER analysis to evaluate the reliability of these suggested methods, like polar coding and noise estimation, in communicating under varying noise levels.

### Noise estimation using optimized-enabled recurrent neural network

Noise estimation using an optimization-enabled RNN is a method for calculating the noise characteristics in a communication system. To achieve correct noise estimates and enhance communication system efficiency, this method leverages the power of recurrent neural networks with optimization techniques. Applications that involve processing time-series information, including signals affected by noise, can utilize recurrent neural networks because of their ability to accommodate data sequences. After training on old data, RNN learns patterns and relationships, enabling it to predict noise characteristics based on incoming signals. Optimization-enabled makes use of optimization techniques to fine-tune RNN’s parameters to enhance its capability for noise estimation. Optimization procedures help the RNN converge on more accurate noise estimates, enhancing nearly all of its capabilities. Based on our research, the use of noise estimation with optimized-enabled RNNs is highly accurate in characterizing noise. This allows our communication system to adjust more effectively under noisy conditions, thereby enhancing the performance and reliability of the proposed approaches.

#### Recurrent Neural Network (RNN)

The RNN is an expansion of the conventional feed-forward neural network, which is designed for processing sequential data. RNN is capable of learning complex and noise characteristics from the sequence which effectively estimates noise and enhances the performance of the Bit Error Rate (BER). RNNs also act like decoders to perform error correction through noise estimation, which is a training network using a backpropagation algorithm to estimate noise levels from the received signals. Over time, this model creates a feed-forward network that enables RNN to learn patterns in sequence by allowing gradient calculation. In this context, we consider an input sequence represented by *M*, a hidden vector sequence represented by *T*, and an output vector sequence represented by *J*. The input sequence is given as $M = (m_{1}, m_{2},...,m_{s})$. A typical RNN computes the hidden vector sequence $T = (t_{1}, t_{2},...,t_{s})$ and the output vector sequence $J = (j_{1}, j_{2},...,j_{s})$ for each position from $g=1,2,...,s$, using the equation 8 and equation 9.

8 $$\begin{aligned} T_{g}=\sigma (N_{mt}m_{g}+N_{tt}t_{g-1}+c_{t}) \end{aligned}$$

9 $$\begin{aligned} J_{g}=N_{tj}t_{g}+c_{j} \end{aligned}$$

In the convention of RNN for Back Propagation Training Time (BPTT), a weight matrix *N* and a bias term *c* are utilized when the function $\sigma$ is a non-linearity function. We do this to process sequence input of different lengths effectively. The BPTT algorithm first trains the model using the provided training data and then saves the error gradient of the output for each step in time. However, training the RNN can be difficult because the gradient can either explode or vanish when trained with the BPTT algorithm.

### Noise estimation enhancements for improved decoding

The research focuses on refining noise estimation procedures to improve the accuracy and efficiency of decoding in communication systems. Noise often accompanies transmission signals, leading to errors during decoding. The main goal of this research is to sharpen the estimates of characteristics for this noise by refining its traditionally used methods to improve a decoding process that helps deliver reliable and accurate information. The goal is to minimize the effects of noise-induced errors and eventually enhance efficiency in communication systems as a whole. This research includes an analysis of the BER-based decoding process. BER refers to the fraction of wrong bits over all transmitted bits and becomes a vital indicator for assessing communication system operation. The BER-based analysis evaluates the performance of decoding techniques in terms of noise and interference handling to provide insight into reliability and efficiency for a communication system.

## Methodology

By adjusting its parameters and configuration, the optimization process enhances the performance of the RNN model. This research developed an optimization method that combines the rapid pursuit patterns seen in Harris hawks with the specific focusing patterns of bald eagles. This combination of properties allows the algorithm to dynamically adapt, learn, and change. As a result, it is an effective and versatile optimization process that can handle complex errors well and provide significantly improved results. This strategy allows the classifier to be fine-tuned for noise estimation. This helps to improve the overall system’s performance. According to Bald Hawk optimization principles, classifier tuning is the process of increasing and refining a classifier’s parameters. This involves adjusting classifier specifications, such as weight thresholds and other fine-tuning features, to increase accuracy. We use Bald Hawk optimization algorithms to iterate the parameter space of the classifier, utilizing the search tricks of Bald Eagles and Haris Hawks.

### Mathematical modeling of proposed Bald Hawk Optimization (BHO) model

BHO is a unique meta-heuristic optimization method inspired by the hunting habits of bald eagles^28^ and Harris hawks^29^. It consists of three essential steps. In the early stages, the bald eagle carefully selects the most favorable location based on the abundance of available food. In the second stage of site search, the eagle hunts in the allocated space for its prey including the exploitation phase of Haris hawk, and in the third stage, it swoops down to find the best place for its prey. The mathematical explanation of BHO model is expressed as follows:

**Step 1: Solution Initialization**

Initially, the solutions are randomly generated based on the hyperparameters including the weights and biases of the RNN model as. The initialization of the hunter population in the proposed algorithm is given using equation 10 as follows,

10 $$\begin{aligned} M = \{M_{1}, M_{2},...M_{z},...,M_{n}\}; 1 <z\le n \end{aligned}$$

where *n* is the number of hunters indicating the solution in population *M*.

**Step 2: Fitness Evaluation**

After initializing the solution, fitness is evaluated for that solution, and the solution with minimum fitness function is indicated as the best solution. In this research, the fitness is evaluated based on minimal Bit Error Rate (BER) using the following equation 11,

11 $$\begin{aligned} Fit(M_{z}^{t+1}) = min(BER(M_{z}^{t+1})) \end{aligned}$$

**Step 3: Solution Update**

The solutions are updated based on the three phases including the search space selection, search, and swooping described below.

**a. Selection of Search Space**

Initially, space of selection is the most important during hunting. The equation 12 is used to generate new positions during this phase:

12 $$\begin{aligned} M_{new}(r) = M_{best}+\beta f (M_{mean}-M(r)) \end{aligned}$$

where, $\beta \in [1,2]$ is the control gain, $f \in [0,1]$ is random number, $M_{new}(r)$ is $r^{th}$ newly generated position, $M_{best}$ is best acquired position during the space of selection, $M_{mean}$ is the mean position, and *M*(*r*) is the most recently generated position. The fitness of each new position, $M_{new}$, will be evaluated, and if it surpasses the fitness of $M_{best}$, $M_{new}$ will replace $M_{best}$ as the new designated best position.

**b. Searching in Space**

After assigning the best search space $M_{best}$, the algorithm updates the position of the eagles within this search space. The updated equation is expressed as below eqquation 13:

13 $$\begin{aligned} M_{new}(r) = M(r)+e(r)(M(r)-M(r+1))+ j(r)(M(r)-M_{mean}) \end{aligned}$$

where, $M_{new}(r)$ denotes the $r^{th}$ newly generated position, while $M_{mean}$ is the mean position. *e*(*r*) and *j*(*r*) are the $r^{th}$ position’s directional coordinates, which can be described as in equation 14,

14 $$\begin{aligned} e(r)= & \frac{ef(r)}{max|ef|};\;ef(r) = f(r).\cos (\theta (r)) \nonumber \\ j(r)= & \frac{jf(r)}{max|ef|};\;ef(r) = f(r).\sin (\theta (r))\nonumber \\ \theta (r)= & b\pi .rand; \; f(r) = \theta (r)K.rand \end{aligned}$$

where $b \in [5,10]$ denoted the control parameter that defines the corner between two points, and $K \in [0.5,2]$ is a parameter that defines the number of search cycles. The new location fitness will be evaluated, and the $M_{best}$ value will be updated based on the results.

**Exploitation phase**

If the pursuit methods of Harris Hawk are integrated with Bald Eagle hunting patterns, there is a definite advantage. Here, the developed optimization method combines Harris Hawks’ tactics of hunting cooperatively and pursuing agilely with Bald Eagle’s superior vision and focused approach to pursuit. The hybridized algorithm would demonstrate collaborative exploitation, where multiple solutions work together to efficiently navigate the solution space with agility, similar to the tactics used by cooperative hawks. In the meantime, the algorithm leverages the highly developed vision of Bald Eagle to identify regions that are susceptible to optimization and employs targeted tactics to pursue optimal solutions. This combination of characteristics allows any algorithm to adapt, learn, and dynamically change strategies, resulting in a more productive and flexible optimization process that can not only cope with the complex optimization landscape but also produce much better results. The hawk pursuit methods and the prey-escaping behaviors are two major parts that make up this phase. Consequently, the goal of this phase is to replicate the hawk’s surprise pounce actions on the victim under investigation. To achieve this objective, we propose two chasing techniques: 1) soft besiege, and 2) hard besiege. In HHO, switching between chasing techniques is determined by two parameters. The following sections describe the proposed strategies:

**Soft Besiege:** In this particular tactic, the concept of soft besiege is applied when both ||*T*||and *d* surpass 0.5. This indicates that the prey is unable to effectively flee as its energy becomes depleted while attempting to evade the hawks and is given by in equation 15, as

15 $$\begin{aligned} Y_{m}(q+1) = \Delta Y(q) - T|RY_{prey}(q)-Y(q)| \end{aligned}$$

**Hard Besiege:** In the field of strategy, two challenging situations arise when the prey’s energy level is very high $i.e. ||T||<0.5$ and the distance between the prey and the predator is relatively large $i.e. d \ge 0.5$. These conditions indicate that the prey is able to effectively flee from its predator. In this scenario, the equation 16 provides the new positions for the hawks.

16 $$\begin{aligned} B = 0.5 Y_{m}(q+1) + 0.5 M_{new}(r) \end{aligned}$$

**c. Swooping**

At this phase, eagles advance towards their intended prey from the optimal position they have acquired. The hunting approach is depicted in equation 17 as follows:

17 $$\begin{aligned} A = 0.5\left( Y_{prey}(q)-Y(q) + rand.M_{best} + jl(r)(M(r)-D_{1}M_{mean}) + el(r)(M(r)-D_{2}M_{best})\right) \end{aligned}$$

Where, *el*(*r*) and *jl*(*r*) are directional coordinates that can be characterized as random numbers from the range [1, 2], while $D_{1}$ and $D_{2}$ also represent random numbers from the same range as in equation 18.

18 $$\begin{aligned} el(r)= & \frac{ef(r)}{max|ef|};\;ef(r) = f(r).\cosh (\theta (r)) \nonumber \\ jl(r)= & \frac{jf(r)}{max|ef|};\;ef(r) = f(r).\sinh (\theta (r))\nonumber \\ \theta (r)= & b\pi .rand; \; f(r) = \theta (r)K.rand \end{aligned}$$

**Termination**

Further, the above steps are repeated until reaching the maximum iteration for optimal solution, denoted as $t < t_{max}$. Finally, the global best solution $M_{best}$ is declared as the best solution for effective parameter tuning.

The Pseudo code for BHO is given in algorithm 1 as follows:

**Algorithm 1:**
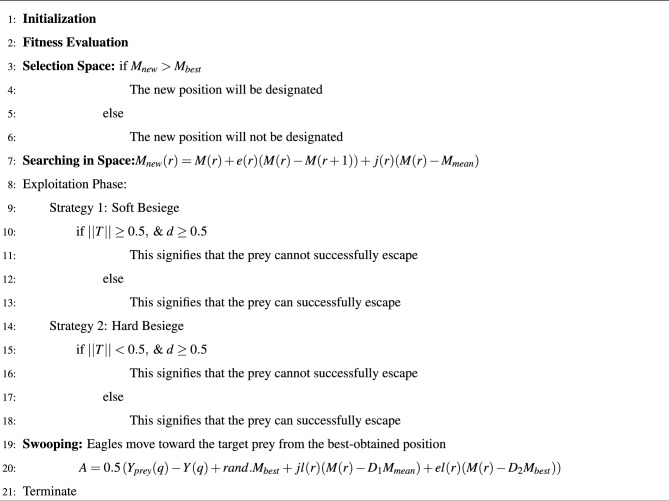
Pseudo code for the BHO model

## Results and discussion

The RNN-based decoder with BHO model is applied to design an efficient polar code construction and compared with alternative techniques.

### Experimental setup

**Table 1:** Summary table of hyperparameters.

**Sr. No.**	**Hyperparameters**	**Value**
1.	Learning rate	0.001
2.	Batch size	32
3.	Activation function	ReLU
4.	Loss function	MSE
5.	Default Optimizer	Adam
6.	Processing range	3.5GHz
7.	Population size	100
8.	Number of epochs	100
9.	Dropout rate	0.5

The experiment validation of the RNN-based BHO model for the polar code construction is implemented using the Matlab Programming Language, the Library is Matlab Deep Learning Toolbox with 8GB internal memory, and Windows 10 as the Operating System. The details of hyperparameters are shown in table 1.

### Performance metrics

**Bit error rate (BER)** BER is the ratio of the number of bit errors to the total number of transmitted bits. The effective polar code decoding required a minimum BER value, determining the high reliability of the decoded data and the effectiveness of the decoding model.

**BPSK BER:** The Binary Phase Shift Keying (BPSK) BER over an Additive White Gaussian Noise (AWGN) channel is the probability that a transmitted bit is incorrectly decoded due to noise. BPSK is a modulation technique where binary data is encoded by the phase of a carrier signal. ratio.

**Frame error rate (FER):** FER is the ratio of the number of frames that contain at least one bit error to the total number of transmitted frames. It focuses on the error rate at the frame level. If the FER value is minimum then the performance of the decoder is high, when dealing with frame-level data.

### Performance analysis

We analyze the performance of the (1024, 512) polar code based on frame error and signal-to-noise ratio (SNR) for Bit Error Rate (BER), Binary Phase Shifting Key Binary Error Rate (BPSK BER), and Frame Error Rate (FER) using an RNN-based decoder with BHO model.

#### Performance analysis based on frame error

**Fig. 2:**
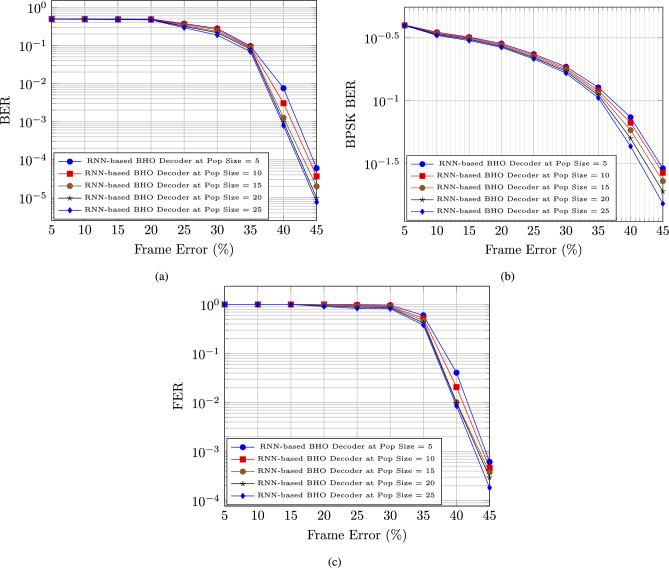
Polar code performance analysis based on Frame error for (**a**) BER, (**b**) BPSK BER, & (**c**) FER.

In figure 2, the performance of the RNN-based decoder with BHO model is evaluated depending on the respective measures as BER, BPSK BER, and FER. The BER for the RNN-based decoder with BHO model is reduced after the maximization of the frame error with the increasing population. The BER for the RNN-based decoder with BHO model for the population size 5, 10, 15, 20, and 25 are 0.0000598, 0.0000365, 0.0000199, 0.00000986, and 0.00000764, respectively at the 45 % frame error, which is in figure 2 a).

The BPSK BER for the RNN-based decoder with BHO model is reduced after the maximization of the frame error with the increasing population. The BPSK BER for the RNN-based decoder with BHO model for the population size 5, 10, 15, 20, and 25 are 0.02900656, 0.026739613, 0.0229692, 0.019018675, and 0.015193137, respectively at the 45 % frame error, which is in figure 2 b).

The FER for the RNN-based decoder with BHO model is reduced after the maximization of the frame error with the increasing population. The FER for the RNN-based decoder with BHO model for the population size 5, 10, 15, 20, and 25 are 0.000614311, 0.000465897, 0.000376094, 0.000295826, and 0.000182007, respectively at the 45 % frame error, which is in figure 2 c).

#### Performance analysis based on SNR

**Fig. 3:**
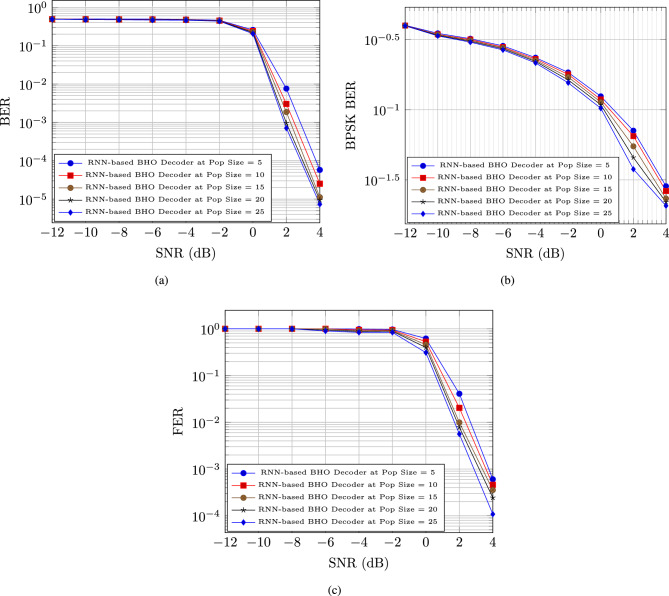
Polar code performance analysis based on SNR for (**a**) BER, (**b**) BPSK BER, & (**c**) FER.

In figure 3, the performance of the RNN-based decoder with BHO model is evaluated depending on the respective measures as BER, BPSK BER, and FER. The BER for the RNN-based decoder with BHO model is reduced after the maximization of the SNR with the increasing population. The BER for the RNN-based decoder with BHO model for the population size 5, 10, 15, 20, and 25 are 0.0000578, 0.0000252, 0.0000112, 0.00000941, and 0.00000735, respectively at the 4 dB SNR, which is in figure 3 a).

The BPSK BER for the RNN-based decoder with BHO model is reduced after the maximization of the SNR with the increasing population. The BPSK BER for the RNN-based decoder with BHO model for the population size 5, 10, 15, 20, and 25 are 0.028500656, 0.026339613, 0.023275062, 0.021974324, and 0.020652839, respectively at the 4 dB SNR, which is in figure 3 b).

The FER for the RNN-based decoder with BHO model is reduced after the maximization of the SNR with the increasing population. The FER for the RNN-based decoder with BHO model for the population size 5, 10, 15, 20, and 25 are 0.000604311, 0.000459897, 0.00035587, 0.000243875, and 0.000108295, respectively at the 4 dB SNR, which is in figure 3 c).

### Comparative analysis

In a comparative analysis, the RNN-decoder with BHO model efficacy is shown using the Polar SC decoder^30^, ESBP based decoder^31^, Bi-LSTM based decoder^32^, Polar BP decoder^33^, Polar SCAN decoder^34^, Polar SSC decoder^35^, Polar SCL decoder^36^, Polar SCL decoder with TLBO, Polar SCL decoder with SARO, Polar SCL decoder with LBR, RNN based decoder with BES and RNN based decoder with HHA.

#### Comparative analysis based on frame error

**Fig. 4:**
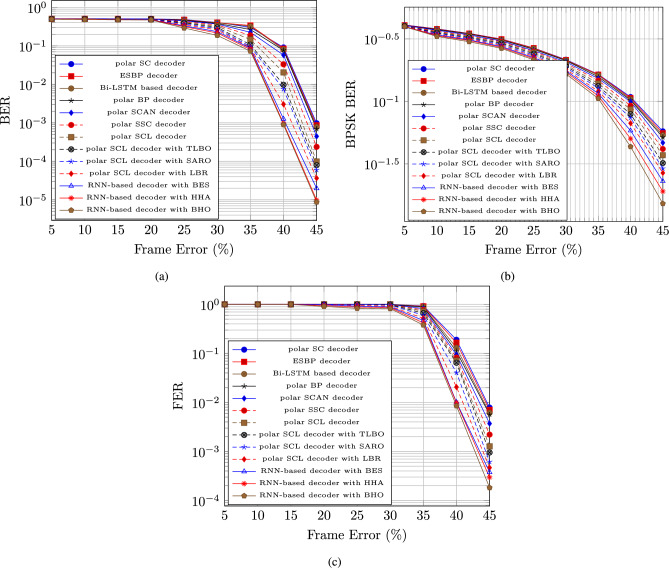
Polar code comparative analysis based on frame error for a) BER, b) BPSK BER, & c) FER.

Figure 4 a) depicts the RNN-based decoder with BHO model BER for polar code construction. The RNN-based decoder with the BHO model surpassed the RNN-based decoder with the HHA model in terms of minimum error, achieving a BER of 0.00000872 at 45% frame error.

The RNN-based decoder with BHO model BPSK BER for polar code construction is shown in Figure 4 b). The RNN-based decoder with the BHO model surpassed the RNN-based decoder with the HHA model in terms of minimum error, with a BPSK BER of 0.015193 at 45% frame error.

The RNN-based decoder with BHO model FER for polar code construction is shown in Figure 4 c). The RNN-based decoder with the BHO model surpassed the RNN-based decoder with the HHA model in terms of minimum error, with a FER of 0.0001820 at 45% frame error.

#### Comparative analysis based on SNR

**Fig. 5:**
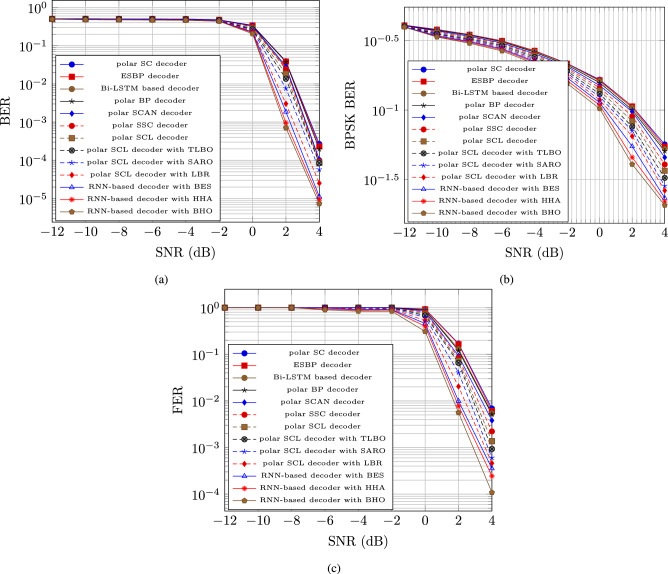
Polar code comparative analysis based on SNR for (**a**) BER, (**b**) BPSK BER, & (**c**) FER.

Figure 5 a) depicts the RNN-based decoder with BHO model BER for polar code construction. The RNN-based decoder with the BHO model surpassed the RNN-based decoder with the HHA model in terms of minimum error, achieving a BER of 0.00000735 at 4 dB SNR.

The RNN-based decoder with BHO model BPSK BER for polar code construction is shown in Figure 5 b). The RNN-based decoder with the BHO model surpassed the RNN-based decoder with the HHA model in terms of minimum error, with a BPSK BER of 0.020652839 at 4 dB SNR.

The RNN-based decoder with BHO model FER for polar code construction is shown in Figure 5 c). The RNN-based decoder with the BHO model surpassed the RNN-based decoder with the HHA model in terms of minimum error, with a FER of 0.0001083 at 4 dB SNR.

### Comparative discussion

In this section, an evaluation is carried out to analyze the performance of various polar code construction models. The Table 2 represents the different models that are being examined. It is important to mention that, when considering the metrics, the RNN-decoder with BHO model exhibits exceptional performance, surpassing every other model. In terms of 45% frame errors, the BER, BPSK-BER, and FER reach their lowest error values of 0.0000087, 0.01519, and 0.000182, respectively. Similarly in the context of 4 dB SNR, the BER, BPSK-BER, and FER achieve values of 0.0000073, 0.02065, and 0.000108, respectively. Compared with other decoder models, the proposed model incorporates the strengths of RNN for noise estimation and introduces the innovative Bald Hawk optimization inspired by the cooperative hunting strategies of Harris Hawks and the focused hunting techniques of Bald Eagles, adaptive polar coding system is developed, which tunes the model to perform effective decoding. RNN is capable of learning complex and noise characteristics from the sequence which effectively estimates noise and enhances the performance of the Bit Error Rate. The collaborative attributes of RNNs and the optimization strategy led to enhanced accuracy in noise evaluation and improved efficiency in decoding processes. These techniques enhance the optimization with robustness and reduce complexity. However, the BHO algorithm assists in improving the performance of polar code generation with a minimal error rate. The experimental results show that the proposed model has a minimum delay with less resource consumption, achieving effective and reliable communication in the presence of noise and other challenges inherent in quantum computing and communication systems.

**Table 2:** Comparative performance for the reviewed and proposed model.

Methods	Frame Error (45%)			SNR (4 dB)		
	BER	BPSK BER	FER	BER	BPSK BER	FER
Polar SC Decoder	0.000977	0.05766	0.007853	0.000268	0.05665	0.006853
ESBP based decoder	0.0008615	0.05565	0.00700	0.0002315	0.05465	0.006058
Bi-LSTM based decoder	0.00078	0.05361	0.00612	0.000207	0.05261	0.005433
Polar BP Decoder	0.000695	0.05164	0.005659	0.000195	0.05064	0.005159
Polar SCAN Decoder	0.000446	0.04663	0.003703	0.000106	0.04563	0.003803
Polar SSC Decoder	0.000238	0.04159	0.002208	0.000094	0.04060	0.002218
Polar SCL Decoder	0.000098	0.03709	0.001271	0.000087	0.03659	0.001371
Polar SCL Decoder with TLBO	0.000079	0.03209	0.000952	0.000072	0.03250	0.000932
Polar SCL Decoder with SARO	0.000060	0.02900	0.000614	0.000058	0.02850	0.000604
Polar SCL Decoder with LBR	0.000036	0.02674	0.000466	0.000025	0.02634	0.00046
RNN-Based Decoder with BES Model	0.000019	0.02296	0.000376	0.000011	0.02328	0.000356
RNN-Based Decoder with HHA Model	0.0000098	0.01902	0.000296	0.0000094	0.02197	0.000244
RNN-Based Decoder with BHO Model	0.0000087	0.01519	0.000182	0.0000073	0.02065	0.000108

### Computational complexity analysis

**Fig. 6:**
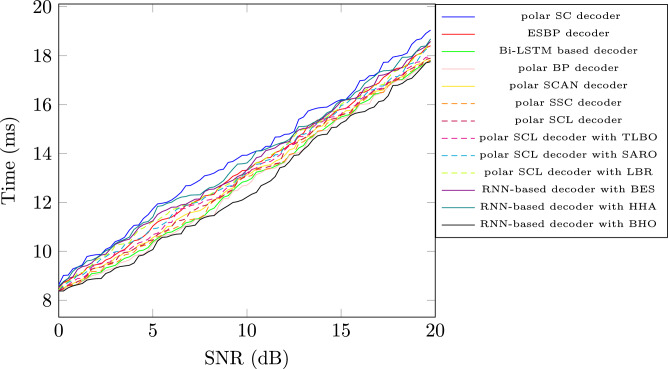
Computational complexity analysis of proposed model with existing models.

The computational time complexity of the proposed RNN-decoder with the BHO model is compared with other decoder models in terms of SNR value to exhibit the complexity of the proposed model. The RNN-decoder with the BHO model achieved a low computation complexity of 17.75ms and other models such as, ESBP based decoder achieved 19.05ms, Bi-LSTM-based decoder 18.40ms, the polar BP decoder 17.79ms, the polar scan decoder is 17.76ms, polar SSC decoder is 17.89ms, polar SCL decoder is 17.82ms, polar SCL decoder with TLO is 17.90ms, polar SCL decoder with SRAO is 17.95ms, polar SCL decoder with LBR is 18.32ms, RNN based decoder with BES is 18.57ms, RNN based decoder with HHA is 18.58ms, and The Polar SC decoder is 18.68ms respectively. Specifically, the incorporation of BHO optimization reduces the computation time by optimally tuning the hyperparameters to attain effective polar code decoding. Figure 6 illustrates the graphical representation of time complexity analysis with SNR value.

### Statistical analysis

Statistical analysis is utilized to determine the patterns in data and concluding those patterns might help to explain the reason for the trial results variation from one experiment to the next. Furthermore, several statistical measures such as best, mean, and variance are computed for the various evaluation metrics. The proposed RNN-decoder with the BHO model achieved a high best value in comparison to other existing models, demonstrating the effectiveness of the suggested model. Tables 3 and 4 depict the statistical analysis of the proposed RNN decoder with the BHO model using the Frame error and SNR based on best, mean, and variance respectively.

**Table 3:** Statistical Analysis of Frame Error.

Models	Metrics								
	BER			BPSK BER			FER		
	Best	Mean	Variance	Best	Mean	Variance	Best	Mean	Variance
Polar SC decoder	0.5048	0.3748	0.0385	0.4090	0.2521	0.0137	1.0000	0.7902	0.1436
ESBP based decoder	0.5044	0.3730	0.0382	0.4075	0.2514	0.0136	1.0000	0.7880	0.1439
Bi-LSTM based decoder	0.5023	0.3673	0.0387	0.4040	0.2492	0.0134	1.0000	0.7807	0.1481
polar BP decoder	0.5040	0.3712	0.0380	0.4061	0.2506	0.0135	1.0000	0.7857	0.1442
polar SCAN decoder	0.5036	0.3696	0.0385	0.4052	0.2496	0.0135	1.0000	0.7824	0.1468
polar SSC decoder	0.5011	0.3650	0.0389	0.4028	0.2487	0.0134	1.0000	0.7790	0.1494
polar SCL decoder	0.5009	0.3609	0.0395	0.4015	0.2481	0.0133	1.0000	0.7679	0.1510
polar SCL decoder with TLBO	0.5002	0.3576	0.0399	0.4000	0.2473	0.0132	1.0000	0.7656	0.1526
polar SCL decoder with SARO	0.4988	0.3564	0.0403	0.3998	0.2470	0.0132	1.0000	0.7610	0.1589
polar SCL decoder with LBR	0.4929	0.3466	0.0396	0.3973	0.2441	0.0130	1.0000	0.7445	0.1616
RNN-based decoder with BES	0.4973	0.3547	0.0387	0.3932	0.2412	0.0130	1.0000	0.7654	0.1527
RNN-based decoder with HHA	0.4975	0.3527	0.0394	0.3986	0.2427	0.0132	1.0000	0.7609	0.1590
RNN-decoder with BHO model	0.4891	0.3439	0.0382	0.3959	0.2400	0.0131	1.0000	0.7445	0.1616

**Table 4:** Statistical Analysis of SNR.

Models	Metrics								
	BER			BPSK BER			FER		
	Best	Mean	Variance	Best	Mean	Variance	Best	Mean	Variance
Polar SC decoder	0.5048	0.3748	0.0385	0.4090	0.2521	0.0137	1.0000	0.7902	0.1436
ESBP based decoder	0.5044	0.3730	0.0382	0.4075	0.2514	0.0136	1.0000	0.7880	0.1439
Bi-LSTM based decoder	0.5023	0.3673	0.0387	0.4040	0.2492	0.0134	1.0000	0.7807	0.1481
polar BP decoder	0.5040	0.3712	0.0380	0.4061	0.2506	0.0135	1.0000	0.7857	0.1442
polar SCAN decoder	0.5036	0.3696	0.0385	0.4052	0.2496	0.0135	1.0000	0.7824	0.1468
polar SSC decoder	0.5011	0.3650	0.0389	0.4028	0.2487	0.0134	1.0000	0.7790	0.1494
polar SCL decoder	0.5009	0.3609	0.0395	0.4015	0.2481	0.0133	1.0000	0.7679	0.1510
polar SCL decoder with TLBO	0.5002	0.3576	0.0399	0.4000	0.2473	0.0132	1.0000	0.7656	0.1526
polar SCL decoder with SARO	0.4988	0.3564	0.0403	0.3998	0.2470	0.0132	1.0000	0.7610	0.1589
polar SCL decoder with LBR	0.4929	0.3466	0.0396	0.3973	0.2441	0.0130	1.0000	0.7445	0.1616
RNN-based decoder with BES	0.4937	0.3526	0.0389	0.3970	0.2408	0.0134	1.0000	0.7654	0.1527
RNN-based decoder with HHA	0.4954	0.3530	0.0390	0.3958	0.2403	0.0135	1.0000	0.7609	0.1590
RNN-decoder with BHO model	0.4906	0.3423	0.0383	0.3958	0.2376	0.0135	1.0000	0.7444	0.1616

### Training and validation loss curve

**Fig. 7:**
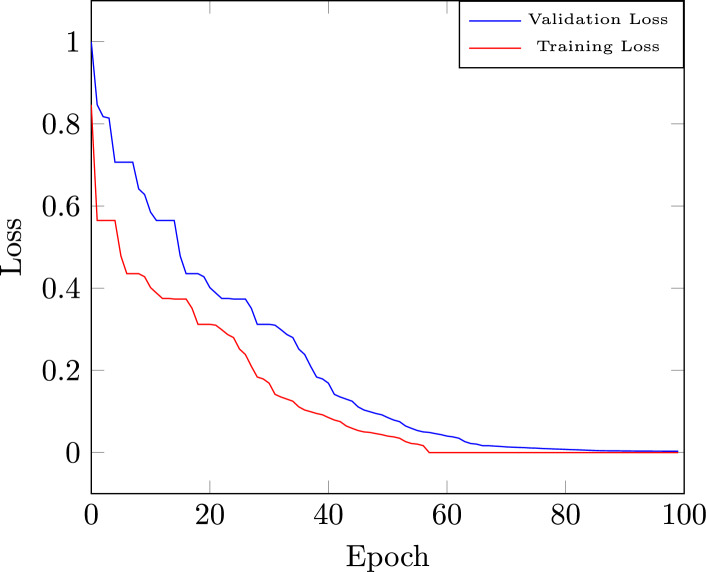
Training and validation loss curve.

Figure 7 illustrates the training loss Curve and validation loss curve of the proposed RNN-decoder with the BHO model, which is plotted against the number of epochs ranging from 0 to 100. The performance of the RNN-decoder with the BHO model decreases from 1 to 0 on training loss and validation loss is decreased from 0.8466 to 0 with multiple iterations. The maximum training loss value that occurred in training data is recorded as 0.12 and decreased over the 10 to 100 epochs. The performance of the proposed RNN-decoder with the BHO model increases based on the minimum training loss and validation loss.

### Latency analysis

**Fig. 8:**
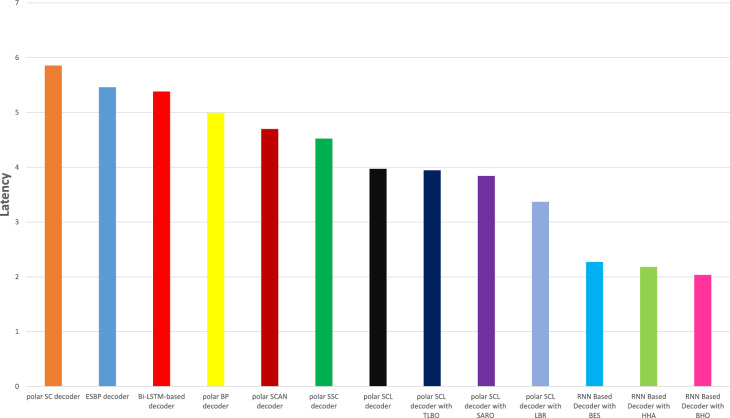
Latency analysis.

Figure 8 illustrates the latency analysis of the proposed RNN-decoder with the BHO model compared with other existing models. Latency analysis explains the time consumption and speed of the decoding algorithm in polar decoding. The proposed RNN-decoder with the BHO model gained less delay of 2.04ms compared with other decoders. Existing models achieved high delay such as the polar SC decoder is 5.86ms, ESBP decoder is 5.46ms, Bi-LSTM-based decoder is 5.38ms, polar BP decoder is 4.99ms, polar SCAN decoder is 4.70ms, polar SSC decoder is 4.52ms, polar SCL decoder is 3.97ms, polar SCL decoder with TLO is 3.94ms, polar SCL decoder with SRAO is 3.84ms, polar SCL decoder with LBR is 3.37ms, RNN based Decoder with BES is 2.27ms, and RNN Based Decoder with HHA gained 2.18ms respectively.

### Memory usage analysis

**Fig. 9:**
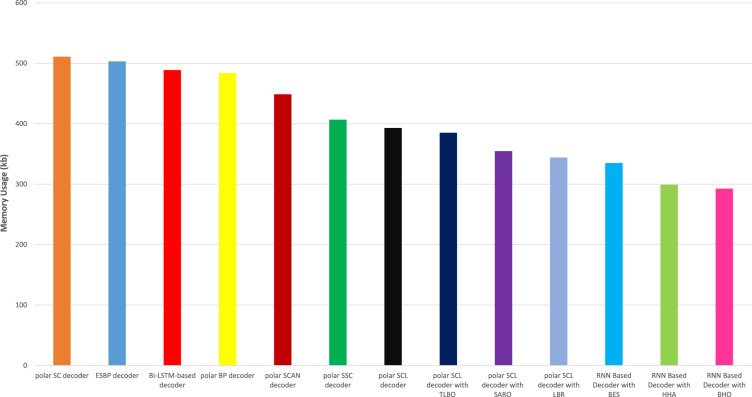
Memory usage analysis.

Figure 9 illustrates the memory usage analysis of the proposed RNN-decoder with the BHO model compared with other existing models. Memory usage analysis is utilized to analyze the memory usage of the proposed RNN-decoder with BHO model with other existing models. The incorporation of RNNs for polar code decoding naturally reduces memory usage. The proposed model used 292.61KB for decoding, and other models reached memory usage of polar SC decoder is 510.93KB, ESBP decoder is 503.06KB, Bi-LSTM-based decoder is 488.70KB, polar BP decoder is 483.78KB, polar SCAN decoder is 448.75KB, polar SSC decoder is 406.61KB, polar SCL decoder is 392.98KB, polar SCL decoder with TLO is 385KB, polar SCL decoder with SRAO is 354.63KB, polar SCL decoder with LBR is 343.99KB, RNN Based Decoder with BES is 334.94KB, and RNN Based Decoder with HHA gained 299.04KB respectively.

## Conclusion

In conclusion, this research makes a significant contribution to the realm of error correction in communication systems, specifically within the context of polar coding. We develop a novel and adaptive polar coding system by combining the strengths of RNN for noise estimation with the innovative Bald Hawk optimization, which draws inspiration from the cooperative hunting strategies of Harris Hawks and the focused hunting techniques of Bald Eagles. The collaborative attributes of RNNs and the optimization strategy lead to enhanced accuracy in noise evaluation and improved efficiency in decoding processes. This approach not only showcases the efficacy of optimization but also underscores the significance of incorporating machine learning techniques for addressing challenges in polar decoding. The findings pave the way for more resilient and adaptable error-correction mechanisms, bringing us closer to achieving effective and reliable communication in the presence of noise and other challenges inherent in communication systems. In terms of 45% frame errors, the BER, BPSK-BER, and FER reach their lowest error values of 0.0000087, 0.01519, and 0.000182, respectively. Similarly, in a 4 dB SNR context, the BER, BPSK-BER, and FER achieve values of 0.0000073, 0.02065, and 0.000108, respectively.

## Data Availability

The datasets used in this investigation are accessible from the corresponding author upon reasonable request at mvraaz.nitw@gmail.com.
